# Male engagement as a strategy to improve utilization and community-based delivery of maternal, newborn and child health services: evidence from an intervention in Odisha, India

**DOI:** 10.1186/1472-6963-15-S1-S5

**Published:** 2015-06-08

**Authors:** Jean Christophe Fotso, Ariel Higgins-Steele, Satyanarayan Mohanty

**Affiliations:** 1 Concern Worldwide US, 355 Lexington Avenue, 19th Floor, New York, NY 10017, USA; 2 United Nations Children’s Fund (UNICEF) Afghanistan, United Nations Complex in Afghanistan, Jalalabad Road, P.O. Box 54, Kabul, Afghanistan; 3 DCOR Consulting Private Ltd, Bhubaneswar, Odisha, India

**Keywords:** Male engagement; Maternal, newborn and child health; Community-based delivery; Odisha, India

## Abstract

**Background:**

In response to persistently poor levels of maternal, newborn and child health (MNCH) in rural India, the National Rural Health Mission (NRHM) was launched to support the provision of accessible, affordable and quality health care in deprived and underserved communities. The Accredited Social Health Activists (ASHAs), local women, are trained as health promoters to generate demand for, and facilitate access to MNCH care in their communities. While they are also expected to provide husbands of expectant women with information on MNCH care and family planning, their reach to the husbands is limited. The aim of this study is to describe the influence of a male engagement project on the utilization and community-based delivery of MNCH care in a rural district of the country.

**Methods:**

We used qualitative data from the evaluation of a project which recruited and trained male Community Health Workers (CHWs) known as Male Health Activists (MHAs) to complement the work of ASHAs and target outreach to men. This paper uses data from in-depth interviews (IDIs) with ASHAs (n=11), Anganwadi Workers (AWWs) (n=4) and Auxiliary Nurse Midwives (ANMs) (n=2); with women who had delivered at home, community health center or district hospital in the few months preceding the date of the interview (n=11); and with husbands of these women (n=7).

**Results:**

Participants’ responses are broadly organized around the facilitation of ASHAs’ work by MHAs, and male engagement activities undertaken by MHAs. More specifically, the narratives reflected gender-based divisions of work and space in three core areas of delivery and use of MNCH services: escorting women to health centers for facility-based deliveries; mobilizing women and children to attend Village Health and Nutrition Days and Immunization Days; and raising awareness among men on MNCH and family planning.

**Conclusion:**

This study sheds light on male engagement as a strategy to improve the delivery, access and uptake of maternal, newborn and child health in the context of prevailing gender norms and gendered roles in rural India. Ultimately, it unveils the complementarity of male and female CHWs in the community-based delivery of, and increased demand for, MNCH services.

## Background

India’s maternal mortality ratio (MMR) declined by approximately 59% between 1990 and 2012, from an estimated 437 per 100,000 live births to 178 per 100,000 live births. Despite the sharp pace of decline in the period between 2006-2012, the country will fall short of meeting the Millennium Development Goals (MDGs) 5 on improving maternal health by 2015 [1,2]. While the trend in under-five mortality rate appears consistent with the MDG 4 target on reducing child mortality, the increase of the share of neonatal deaths in under-five deaths from 41% to 55% between 1990 and 2012, remain a cause of concern [2,3]. Equally important are the wide geographical disparities that persist in India’s maternal, newborn and child health (MNCH) indicators. The most recent estimates suggest that MMR varied from as low as 66 per 100,000 live births in the State of Kerala to 235 in Odisha and 328 in Assam. Further, under-five mortality remains more than 80% higher in rural India than in urban areas [1,4,5].

In response to persistently poor levels of maternal and child health in rural India, the National Rural Health Mission (NRHM) was launched in 2005 as a framework for the provision of accessible, affordable and quality health care in deprived and underserved communities in rural areas [6-8]. At the center of the Reproductive and Child Health (RCH) Program which is run under the umbrella of NRHM, are the Accredited Social Health Activists (ASHAs), local women trained as health educators and promoters to generate demand for, and facilitate access to MNCH care in their communities. Other related initiatives promoted under NRHM include the management of malnutrition through Village Health and Nutrition Days (VHNDs), and the system of free transport of pregnant women to health facilities through the Janani Express Yojana program [9,10]. The recent increase in institutional delivery in India has been largely attributed to the introduction of ASHAs [11,12]. Acknowledging the central role of men in women’s reproductive health, the RCH program includes the training of health workers to provide husbands of expectant women with information on MNCH care and family planning [4,6].

### Male engagement in MNCH: rationale and implications

In India and other parts of the developing world, gender-based power inequalities in reproductive health decision-making have been acknowledged as a fundamental constraint to women’s access to reproductive health services, and ultimately, a barrier to improved health outcomes [13-15]. In these settings, women are largely dependent on their husbands for health-related decisions, making the behavior, knowledge and attitudes of men an integral element of the reproductive health status of the family [15,16]. Men also resort to making decisions about their wives’ health care as a consequence of women’s structural and cultural dependence on men, due to limited mobility and limited educational and economic opportunities for women [17]. According to India’s 2005/06 National Family Health Survey, the main reason pregnant women did not make antenatal care (ANC) visits or did not deliver in a health facility was that their husbands did not think it was necessary or did not allow them to do so. Nationally, only 40% of pregnant women attend ANC visits and only 45% deliver under the supervision of a skilled health personnel. The report concluded that men’s participation in maternal health care should be strengthened, and the information provided to men more comprehensive [18]. Another study on the same topic argues that the formulation of programmatic and policy interventions related to increased male involvement in women’s health is still in its infancy, partly due to mixed findings from existing research [19].

The program of action developed at the 1994 International Conference on Population and Development (ICPD) emphasized the need for equity in gender relations, especially men's shared responsibility and active involvement to promote reproductive and sexual health [16,20]. Even prior to this development, there was a recognition that men constituted an important, yet untapped resource in efforts to improve the health of women and children [19], and through abuse or neglect, their actions had direct consequences on the health of their wives and children [21,22]. Overall, husbands’ social support and perceived social norms were identified as underlying factors associated with delivery care utilization [15,23]. As a result, there was a paradigm shift after the ICPD meeting from “men as clients” to “men as partners.” The former concept entails addressing men’s reproductive health needs, while the latter emphasizes the central role men play in supporting women’s health, and implies recruiting men and raising their awareness about danger signs in labor, transportation plans, the benefits of family planning for women’s health, among other topics [20].

Many studies have shown that well-designed male involvement programs have the potential to generate changes in men’s attitudes and behaviors [17,21,22]. However, a number of obstacles to male involvement in maternal and child health have been identified, the most critical of which is the poor knowledge of husbands and other members of the family on the do’s and don’ts during pregnancy, child birth and the postpartum period. Other frequently cited barriers include social stigma, shyness and embarrassment, work obligations, and poor communication between husbands and wives [24,25]. Even when Indian men have positive attitudes towards MNCH care, the family environment characterized by the interference of mothers-in-law [14,15,19], may not favor the provision of care to pregnant women, compromising access to good quality home-based or facility-based care [14,17,19]. Recommended strategies to address these barriers include engaging in communication on appropriate care during pregnancy, child birth and the postpartum period, as well as targeting and equipping men on appropriate home-based care, preventive care and danger signs through effective counseling [21,22].

**Figure 1:**
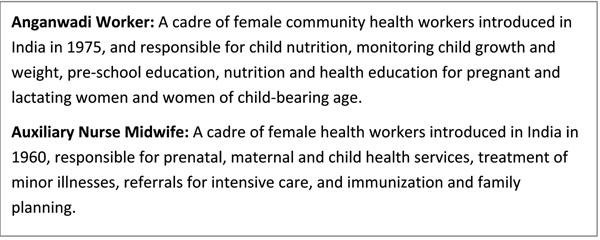
Anganwadi Worker and Auxiliary Nurse Midwife

The role of community health workers to counsel and provide information on reproductive and child health is well recognized. In the context of rural India, the ASHA, Anganwadi Workers (AWWs), and Auxiliary Nurse Midwives (ANMs) are women who initiate and maintain a dialogue with mothers and other women in the community, provide health information and facilitate referrals to health facilities [26,27]. Figure 1 presents a brief description of AWWs and ANMs. Studies conducted in Odisha State have revealed that ASHAs and AWWs are the primary providers of health and nutrition information in the community; they have strong credibility with community members, but are poor at interpersonal communication and counseling [28,29]. Being female, their reach to the male members of the community is limited, as evidenced by the evaluation of the ASHA program conducted in eight states of India [30].

The aim of this study is to describe the influence of a male engagement project on the utilization and community-based delivery of MNCH care in a rural district of India. Specifically, the research questions guiding the analysis are: To what extent did male CHWs complement the work of their female counterparts and fill important gaps in community MNCH service delivery? What is the perceived influence in the community of male CHWs’ engagement with men on the utilization of MNCH services?

## Methods

### The Male Health Activists project: overview

**Figure 2:**
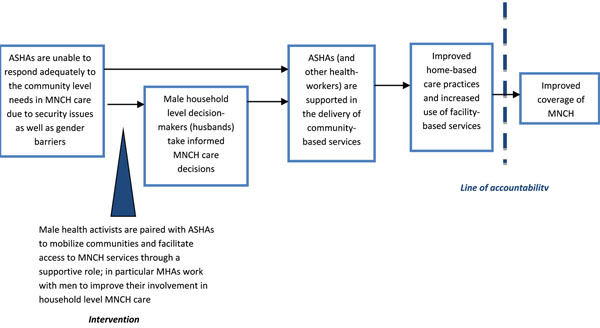
The intervention’s theory of change

The Male Health Activists project, implemented in the district of Keonjhar in the State of Odisha, was designed to address some of the challenges ASHAs face in delivering their services, in particular encouraging men to take a more active role in the health of mothers and children. The project recruited and trained male community health workers known as Male Health Activists (MHAs) to complement the work of ASHAs and target outreach to men as a way to extend community-based delivery of health services for women, neonates and children. The aim of the project was to improve the coverage of MNCH services delivered by the formal health care system, and improve home-based management of MNCH and care-seeking for prevention and treatment services. The project’s theory of chance is depicted in Figure 2.

The State of Odisha is among the six states (out of the 29 that make up India) with the highest rates of maternal and child deaths. Approximately 37% of Odisha’s population live below the poverty line. Keonjhar district, the location of the intervention, has some of the worst MNCH indicators and ranks 24^th^ out of the 30 districts in the State on the Human Development Index [31]. The district is one of the 250 districts in the country receiving special funds for underdevelopment status. In terms of health outcomes, 12 out of the 13 sub-district administrative areas of Keonjhar are classified by the NHRM as ‘high focus’ due to poor MNCH performance, with two of these (Banspal and Harichandanpur) considered by the government as the most difficult in terms accessibility and low service utilization.

The MHA project was implemented in a total of 205 villages in six out of the 13 blocks of the districts, representing a total population of about 600,000 [32]. The pilot was launched in February 2011 for a period of approximately two years. A total of 205 MHAs (one per village, on average) were recruited through a selection process adapted from the NRHM’s guidelines for selecting ASHAs. Selection criteria emphasized characteristics and skills associated with responsibilities related to health promotion and linkages to facility-based care. In consultation with health authorities and community leaders, the project team sought men who were middle-aged, married, had a minimum level of education (completion of primary school), and were well-perceived in the community. In this paper, we use the terms MHA and male CHW interchangeably, as we do for ASHA and female CHW.

Before beginning work in communities, MHAs were trained using the NRHM’s ASHA training modules. They were then paired up with ASHAs to conduct the following activities:

• Work with, and support ASHAs at the village level in the referral of women and children to facility-based care;

• Coordinate with other community-level health workers for the delivery of other outreach and community-based reproductive, maternal, newborn and child health (RMNCH) services (e.g. antenatal care, immunization, contraception);

• Counsel husbands/fathers on RMNCH health issues and encourage appropriate home-based care and care-seeking;

• Work with other village-level leaders to support the planning and implementation of other health-related activities at the village level.

### Data source

This study used data from the evaluation of the MHA intervention, which relied primarily on endline qualitative investigations. Data collection took place in November and December 2012. Specifically, we used data from in-depth interviews (IDIs) with ASHAs (n=11), AWWs (n=4) and ANMs (n=2); with women who had delivered at home, community health center or district hospital in the few months preceding the date of the interview (n=11); and with husbands of such women (n=7). A purposeful selection of ASHAs, AWWs and ANMs was undertaken, ensuring inclusion of health workers from all blocks and a range of villages based on distance to the block headquarters. Women were selected from the delivery records of three community health centers and the District Hospital (two women per facility). Names of respondents were drawn randomly from the Labor and Delivery Register for the period of September to November 2012, after sorting the records by project or non-project villages. Additionally, three women who had recently delivered at home were selected in the catchment areas of these four facilities. The same procedure was used to select the women whose husbands were to be interviewed. MHAs were also interviewed, but their perspectives were deemed less critical to the achievement of the study’s objectives, as they were the target of the intervention, and had been recruited, trained and utilized by the project.

The interviews with women and men were designed to explore knowledge, attitudes and behaviors related to RMNCH and to understand MHAs’ involvement and the support (or lack thereof) they provided directly to community members considered the targets for these types of interactions. The interviews with ASHAs, AWWs and ANMs sought to understand the role and relationship of MHAs vis-à-vis other health workers and to explore the type and extent of support provided by MHAs. Informed consent was obtained from each respondent after describing the study objectives. Ethical approval was obtained from the ethics committee of the Department of Health and Family Welfare of the Government of Odisha.

### Data collection and analysis

All ASHAs, AWWs and ANMs, women and men were interviewed in their respective homes by locally-based researchers who used pre-tested semi-structured guides in the local language (Odiya). Each interview involved two researchers, one conducting the interview and the other in charge of note taking and audio-recording. Responses were audio-recorded, transcribed verbatim and translated to English. Data coding and analysis was conducted manually. Themes related to the study’s objectives were identified from the interview guides. The transcripts were then read several times and tabularized along these themes, and new themes emerging during the analysis were incorporated. The analysts paid attention to variations in reports within and across types of respondents. The data for this paper are based on the following themes: Challenges and difficulties faced with access to and provision of MNCH care; Opportunities for increased access to, and provision of MNCH care; Perceived roles of MHAs; and Positive and negative aspects of MHAs’ work.

## Results

### Study participants

Table 1 shows the distribution of the ASHAs, AWWs, ANMs, women and men interviewed by block. All ASHAs interviewed were married, had the required educational qualification of Class 8 or above as stipulated by the NRHM guidelines, were mostly in the 30-45 year age range, and started working in their current jobs between 2006 and 2008. All of the AWWs and ANMs interviewed were married, and had been in their positions for at least a decade.

**Table 1:** Distribution of study respondents by block

Block name	ASHAs	AWWs & ANMs	Mothers	Fathers	Total
Banspal	2	1	3	2	8

Champua	3	1	1	0	5

Ghatagaon	2	2	2	2	8

Harichandanpur	2	0	2	1	5

Joda	1	1	2	0	4

Telkoi	1	1	1	2	5

Total	11	6	11	7	35

Of the 11 women respondents, five were aged between 20 and 24 years and three under the age of 20. For nearly half the women, this was their first child. The majority of women had no formal education, as the intervention villages included some of the least developed areas of the district. Almost all women interviewed reported agricultural work or wage labor as their main occupation. On average, the men selected for interviews were older and more educated than the women. They were mostly drivers, agricultural workers, or self-employed (e.g. bicycle repair), or employed in factories/mining – a profession which entails living away from home for part of the week.

From the analysis, participants’ responses were organized broadly around the facilitation by MHAs of ASHAs’ work, and male engagement activities undertaken by MHAs. More specifically, the narratives elicited from the respondents reflected gender-based divisions of work and space in three core areas of delivery and use of MNCH services: escorting women to health centers for facility-based deliveries; mobilizing women and children to attend Village Health and Nutrition Days and Immunization Days; and raising awareness among men on MNCH and family planning. Respondents’ views tended to assign certain activities and practices mainly to one gender or the other.

### Escorting women to health centers for facility-based deliveries

Almost all respondents pointed to MHAs’ facilitation of women’s access to health centers, especially at night, and to support provided while at the facility. Most women interviewed spoke about the risks associated with night deliveries in a context of long distances to facilities and lack of readily available transportation means. They acknowledged the limitations of female CHWs in this regard and welcomed the introduction of male CHWs. “*In the night ASHA Maa [ASHA] can’t go anywhere*, *ASHA Bapa [MHA] is Purusha [meaning male] so he can go*, *he can help everything*,*”*, relayed a woman in Telkoi who recently had a facility delivery. Another woman from Banspal, in response to a question on the relevance and usefulness of MHAs and whether they should be maintained beyond the life of the project added: *“Because in the middle of the night they will go to the hospital no matter how far it is*, *or will make phone calls. In the night he can go running for the patient”*

The narratives from virtually all ASHAs also pointed to the crucial roles played by MHAs around and during delivery, especially at night when they facilitate transport and provide security, a role that female CHWs were not able to play due to security concerns. An ASHA from the Banspal block, regretting the lack of support from husbands noted: “*If a delivery goes in the night then*, *previously I was going alone; the road is full of jungle*, *can we take our husbands all the time? If he [MHA] is there*, *I do not feel scared.”*

While accompanying women and their children at night to the facilities was seen as a critical function, women and ASHA respondents also valued MHAs’ support with regard to transport. Most of the intervention villages are located in forest and hilly areas and are not connected with motorable roads, making it impossible for the Janani Express Yojana (the free transport system provided under the NRHM framework) or private vehicles to reach pregnant women and transport them to facilities for delivery. The interview results suggested that the MHAs support ASHAs by making local transportation arrangements to carry pregnant women from their homes to a place where vehicles can reach them. Commenting on the benefits of a male CHW, a woman in the block of Ghatagaon stated: “*with ASHA Bapa [MHA]*, *vehicles and other things will be done’*. Her peer from Harichandanpur was more assertive, illuminating the gender norms about community service delivery and pointing to possible constraints faced by female CHWs in relation to their own childbearing needs:

*“He [MHA] is coming in the middle of the night and then wherever there is a problem, if for the vehicle he phones and didn’t get, then he goes by walk, then taking the patient and reaching there. And the female ASHA is having two small children, and being a female she is unable to go in the night.”* (Woman in Harichandanpur).

An ASHA interviewed in Banspal shared these perspectives and added: “*if a pregnant woman starts getting pain he can call me and call the Janani Express Yojana vehicle and take her.”*

Men respondents were keen to admit that MHAs were indeed filling important gaps related to access to facility-based care for women and children. Both the men and women in the villages recognized the importance of male CHWs, most of their narratives pointing to the prompt and untiring efforts by MHAs to solve problems, including cycling to various places in search of solutions:

*“ASHA Maa cannot do the work like ASHA Bapa [MHA]. Suddenly there is some work, by chance if they can’t get the Janani Express Yojana vehicle, if ASHA Bapa is there then he can cycle down to Ghatagaon or can arrange a vehicle from another village. ASHA Maa, what can she do?”* (Man in Ghatagaon).

Once at the facility, female and male CHWs’ roles were seen by men as complementary. A man in Telkoi explained: *“Suppose there is any problem or danger*, *he will catch the ASHA and escort to the medical. If you go to medical taking both of them then it becomes very easy.”*

Division of labor along the gender lines emerged even more strongly from the narratives with regard to actual service delivery at the facility. The data indicate that once at the facility, MHAs handled some tasks outside of the delivery room as needed, which ranged from keeping track of the family’s personal items, obtaining medicines, and in cases where a blood transfusion was necessary, acting as an advocate to obtain donated blood. “*Male ASHA [MHA]*, *he is a male; how can he touch the female? He does all other work like getting the medicine and other things which the Doctor writes*, *he gets those things*, *and shops are bit far.”* Besides the logistics, engaging with husbands around delivery was seen as a major contribution of male CHWs:

*“He [MHA] cannot enter in to the delivery room. He brings the medicine which is required and all things he [the health professional] tells; he [MHA] tells the husbands. I can convince the mothers but not the husbands”* (ASHA in Champua).

Women’s narratives clearly emphasized the roles of MHAs during delivery, including filling important gaps due to husbands’ and family members’ poor support and birth preparedness. A mother in Harichandanpur who had recently delivered at a facility noted: *“Jhia ASHA stayed near me*, *and Purusha ASHA went out and then oil*, *soap*, *the things which is given*, *went running to get those.”* The presence of a male CHW is critically needed in this instance, especially since, as stressed by another respondent, shops and pharmacies may be quite far from the facility.

### Mobilizing women and children to attend Village Health and Nutrition Days and Immunization Days

Respondents’ narratives also acknowledged the facilitative roles of MHAs in the planning and implementation of Village Health and Nutrition Days (VHNDs) and Immunization Days. Village Health and Nutrition Days and Immunization Days in settings like Keonjhar District are the centerpiece of community-based MNCH service delivery. Services provided at these monthly health events run by ASHA, AWW and ANM include registration of pregnant women, antenatal care (ANC), immunization, growth monitoring of children under the age of five years, distribution of contraceptives, provision of drugs to patients as required, and supplementary nutrition to pregnant women [30]. Awareness-raising for high turnout on the day of services is typically conducted by CHWs prior to and the morning of the outreach activities. In their narratives on the roles of the newly introduced male CHWs, ASHAs identified tangible constraints that could only be addressed by MHAs. One of the ASHAs we interviewed in Banspal told us:

“Now the far away sahi is covered. There is a jungle in the middle way; the far away village is atop the hill and a single woman alone can’t go. If any child is left out for the polio immunization then we four, two Anganwadi Didi and two ASHA Didi go together because there is a jungle. But he [MHA] goes alone by cycling and keeps the cycle in the mid-way and climbs the hill.”

Even in easier-to-reach places, the role of MHAs in community mobilization was commendable, judging by the answers we received. In Champua for example, an ASHA respondent admitted: *“I go calling in the morning of the VHND and immunization Day; the children have not come or making late or may be due to some reason not coming then*, *he [MHA] goes 2 to 3 times by cycle to call them. Suppose I see someone has not brought her child then he goes again and brings the child.”* An AWW in Ghatagaon was more emphatic: *“We have many households here and there; he [MHA] being a male goes by the cycle and calls them. We girls cannot go to all the places*, *so he can cycle all the houses and get the reports.”*

While female and male respondents did not acknowledge the relevance of MHAs in VHNDs and Immunizations Days as strongly as the female CHWs, they noted, as a woman respondent in Joda, that *“They [MHAs] also come to home for calling for meetings. Meetings used to be done there*, *so he comes for calling.”*

### Raising awareness among men on MNCH and family planning

Our respondents largely viewed the advent of MHAs as the opportunity to engage with men on family planning and MNCH issues. Women and ASHA respondents viewed the presence of male CHWs as an opportunity to engage with men on matters related to RMNCH. Most female CHWs interviewed pointed to increased engagement of MHAs with men, which to some degree, resulted in positive behavior change. An ASHA in Banspal described: “*After his appointment*, *the husbands who do not understand*, *whatever we say they avoid and shout at us. He [MHA] convinces the males more. Men used to say that ASHA is coming and misguiding our wives. But the MHA makes them sit and he tells them that it is for your good only. Whenever he does they understand.”* To stress the gender dynamics in that society, she added: “*When I was going to that sahi alone*, *they used to shout at me. If the Purusha ASHA [MHA] is there and I reach there*, *no one shouts or anything*, *rather they will talk and listen nicely.”*

The interviews with men did not seem to be as unequivocal on MHAs’ contributions. In general, most women reported not knowing if MHAs talked to their husbands or not; the ones who reported being aware of the engagement said they did not know the subject discussed between the MHAs and their husbands. Men on the other hand, seemed too busy with work and other activities, and as a result, did not pay close attention to the invitations made by MHAs to discuss MNCH issues in group or individual meetings. The only male respondent to acknowledge the engagement by MHA told us the advice on birth preparedness: *“He told us to keep money in hand. By chance if there is any problem*, *the vehicle and all*, *so we should keep the money properly.”*

The specific work of MHAs on contraceptive use among men also emerged from female CHWs’ narratives, and less so from women’s and men’s. Cognizant of the burden and consequences of high fertility in these poor communities, many ASHAs expressed the wish that men in their catchment areas be more open to, and accepting of contraceptive use. In Champua, one of the ASHAs we interviewed said: “*If he [MHA] can convince the males. The main thing is if Purusha [husband] understands then they can convince their wives about the so many children.”* Some of the ASHA respondents cast a somewhat optimistic view regarding uptake of contraception among men. Some of the ASHAs, AWWs and ANMs interviewed spoke of door-to-door meetings conducted by MHAs, which they said are now convincing the men on contraceptive use. One responded noted:

*“For the Purusha swasthya Bahini meeting, mainly the condoms were being used; he [MHA] is making it easy for the condom; he conducts the meetings and convinces the men. I could not do with condom; I give them to him [MHA] and he does what is needed.”* (ASHA in Telkoi).

Whether or not the project resulted in contraceptive uptake among men, almost all respondents hinted that strategies to increase contraceptive use will need to incorporate male CHWs’ engagements with men:

*“Look, a thing like an operation, husbands used to shout on us that how can these girls work with us. ASHA Bapa [MHA] can tell to the husbands to allow their wives to undergo the operation [sterilization]. We cannot tell the males, only ASHA Bapa can and men can understand properly. What I was not able to say, that can be said by him.”* (AWW in Champua).

Besides the gendered division of labor between which emerged as a constant theme from the narratives, the idea that the newly recruited MHAs provided another set of hands regardless of sex was also apparent from the interviews with female CHWs. In these instances, MHAs were reportedly working as directed by the ASHAs, the health workers with longer experience working in the communities. One ASHA in Harichandanpur reported: “*We divide the areas between us to bring the children during immunization.”* In other instances, one CHW will take the responsibility when the other is otherwise occupied, as illustrated by this report from one ASHA in Telkoi: “*He can work when I am absent. Recently a delivery took place; MHA took the whole responsibility because at that time I was on training. About 2-3 cases he has handled alone.*”

## Discussion

Efforts to provide accessible, affordable and quality health care in India’s deprived and underserved communities through the National Rural Health Mission (NRHM) notwithstanding [8,11], most parts of rural India remain characterized by worrying high levels of poor maternal and child health indicators. While the introduction of ASHAs and other interventions designed under the NRHM have undoubtedly contributed to the recent improvements [12,26,29], progress remains hampered in rural and remote settings by lack of men’s understanding of, and interest in the health of mothers and children [6,19].

The findings of this study shed light on two major, gender-related constraints ASHAs face in their duties. First, MNCH care seeking behaviors in most rural societies are often guided by deep rooted socio-cultural and traditional beliefs, superstitions, myths and misconceptions that can be hazardous to health [6,12,24], and are characterized by imbalanced and unequal decision-making favoring men [13,15]. Indeed, rural women do not only face barriers in accessing health services linked to poverty and illiteracy, they also face challenges due to the lack of control of their reproductive health and other health matters [5,24,25]. Second, our intervention area is characterized by challenging terrain of hills and rivers that hinder the development of road networks, which further limits the development of an emergency obstetrics transportation system [10,17]. This type of environment makes the work of female CHWs more difficult. Surprisingly, no study to our knowledge has investigated the implications of these constraints for female CHWs’ duties and responsibilities in the context of India’s NRHM. Evidence from Rwanda, one of the few countries with male-female pairs of CHWs, indicates that these pairs may be helpful in settings where it may not be safe or socially acceptable for women to travel alone [33].

Escorting pregnant women and women in labor to health facilities is a critical component of efforts to improve the uptake of facility-based MNCH care in contexts like rural India. Our results indicate a division of labor for CHWs along gender lines. As women, ASHAs’ work can be limited by their mobility, especially at night or through the hilly and forest areas. The predominantly favorable views from ASHAs on the contribution of male CHWs, all pointing to the complementarity of both genders, are noteworthy, especially with regard to accompanying patients in transit. From ASHAs’ and women’s narratives, this division of labor continued to hold at the health facility during and around the time of delivery. The presence of a male CHW helped the ASHA to remain focused on the delivery, while the MHA promptly dealt with logistics like obtaining an admission ticket, buying medicines, and arranging for blood transfusions when needed. These joint efforts ultimately result in improved access to services and possibly better quality of care in some areas while at a health facility.

According to men and ASHAs interviewed, the coverage of MNCH services improved as the male CHWs improved outreach in a difficult geographical set-up, sharing the work with their female counterparts. Apart from arranging transport and accompanying pregnant women in emergency cases, ASHAs appreciated the support provided by the male CHWs who often did not spare energy and time to walk or cycle, and sometimes climbed hills to reach households in different settlements.

Enlisting the support of men is the necessary step and perhaps a pre-condition to improved health for women, newborns and children. As widely acknowledged, despite India’s significant advances towards gender equality achieved among some groups, the majority of women still suffer from the effects of extreme inequality and weak position, as evidenced by the country’s ranking of 112 out of 134 countries on the global gender gap index [5]. The global gender gap index measures the relative gaps between women and men across four key areas: health, education, economy, and politics. As a consequence, non-use of MNCH services is partly explained by the opposition of husbands, due to limited knowledge about healthy behaviors and actions to take during pregnancy, delivery and the postpartum period [14,15,19,24]. The design of the intervention covered in this paper rests on the premise, as reported in many studies, that men should also be target audiences for women’s reproductive health interventions through specially designed channels and messages, given their low knowledge levels and the imbalance in decision-making between men and women in many societies [20,24,25,34].

Our findings tend to suggest that while engagement with men was regarded as a key component of ASHAs’ work [26,27], the challenges associated with gender dynamics in Indian society were largely neglected. The ASHAs we interviewed welcomed the addition of male CHWs as partners in creating awareness among and motivating men to embrace positive behaviors and supporting roles regarding the health of their wives and children, including on sensitive issues such as use of family planning services. Most women and female CHWs interviewed expressed confidence in male CHWs’ engagement to reduce the uninformed or resistant behavior of men towards use of family planning and MNCH services. These findings, and particularly female CHWs’ and women’s invocation of the benefits of MHAs’ engagement with husbands, challenge the persistent rhetoric on Indian men’s involvement in MNCH and reproductive health. In their study in Rajasthan, India, Karol et al. found ASHAs had rather weak knowledge with regard to motivating men and women in the community to adopt healthier reproductive behaviors including use of family planning [27].

Our interviews with community men did not indicate that MHAs’ engagement with them was effective at improving men’s knowledge on MNCH and family planning. One possible explanation for this finding may be that men perceive other priorities such as remunerated activities as more important in their daily lives than engaging with a male CHW on MNCH and family planning, especially if the issues are not emergency-related matters for the family. Additionally, as male CHWs had newly assumed their role and a refresher training occurred toward the end of the project, they may not have refined techniques to engage men in conversations related to preventive and curative care for their wives and children. Additionally, evidence is emerging on the usefulness of peer support networks among CHWs, which is evidenced by the ways in which the ASHAs and MHAs collaborated. A recent study indicates that social support mechanisms for CHWs are important for improving care and information that CHWs provide [35].

At the core of the NRHM is the balance between facility-based and community-based service delivery, the latter being linked to VHND and Immunization Days, both events requiring strong engagement of ASHAs [11,27,29]. This study suggests that as with the escort of women to health facilities, gender and peer support dimensions associated with the effective delivery of these community-based interventions seemed to have been underestimated in the design of the ASHA program. An overwhelming majority of narratives from female CHWs, women and men pointed to improvements brought about by the recent introduction of male CHWs. Most respondents noted that several logistical problems associated with the planning and implementation of village outreach activities can only be adequately handled by male CHWs alongside ongoing work of female CHWs.

## Limitations

While the study focuses on the perspectives of health workers who experienced the MHA intervention and community members with relevant profiles to be targeted for support by CHWs, the lack of baseline data, and a possible bias in recalling the situation that prevailed before the introduction of male CHWs by the project are important limitations. Despite these limitations, the study has shed light on challenges overlooked by the NRHM framework.

## Conclusion

Building on the success of the ASHA program of delivering community-based information and care to families in rural communities in India, this study sheds light on male engagement as a strategy to improve the delivery, access and uptake of maternal, newborn and child health in the context of prevailing gender norms and gendered roles in the health care sector [16,20,36]. Our findings provide important new insights regarding barriers that continue to limit the scope and reach of the ASHA program, and unveils the complementarity of male and female CHWs in the delivery of, and increased demand for, MNCH services. The findings on the facilitative roles of MHAs suggest including male CHWs as a part of the NRHM’s model for increased delivery of, and demand for MNCH services. It is important, however, to acknowledge that female CHWs are performing important roles in challenging locations to improve MNCH outcomes [37]. The introduction of male CHWs to reinforce these successes, as shown by this study, should operate in ways that do not contribute to widening gender inequalities in favor of men that are rampant in many rural communities in the developing world.

## Authors’ contributions

JCF conceptualized the study; JCF and AHS conducted the literature review; SM coded the data; JCF led data analysis; JCF, AHS and SM all contributed to the writing of the paper, and read and approved the final manuscript.

## Authors’ information

JCF is Associate Director of Research, Monitoring and Evaluation at Concern Worldwide US; AHS is Knowledge Management Specialist at UNICEF Afghanistan Country Office; SM is Director at DCOR Consulting, India.

## Competing interests

The authors declare no competing interests.

## Declarations

The publication costs for this article were funded by the *Innovations for Maternal*, *Newborn & Child Health*, an initiative funded by Bill & Melinda Gates Foundation.

This article has been published as part of *BMC Health Services Research* Volume 15 Supplement 1, 2015: Improved access to maternal, newborn and child health services: strengthening human resources for health. The full contents of the supplement are available online at http://www.biomedcentral.com/bmchealthservres/supplements/15/S1.
